# Distinct experiences and care needs of advanced cancer patients with good ECOG performance status: a qualitative phenomenological study

**DOI:** 10.1186/s12904-024-01425-3

**Published:** 2024-04-17

**Authors:** Ping Chen, Mingfu Ding, Changlin Li, Yujuan Long, Deng Pan, Li Ma, Taiguo Liu, Cheng Yi

**Affiliations:** 1 Abdominal Oncology Ward, Division of Medical Oncology, Cancer Center, West China Hospital, Sichuan University, No. 37 Guoxue Alley, Wuhou District, Chengdu, 610041 Sichuan Province China; 2 Rehabilitation Medicine Department, West China Hospital, Sichuan University, No. 37 Guoxue Alley, Wuhou District, Chengdu, 610041 Sichuan Province China; 3 Department of Oncology, Chengdu Seventh People’s Hospital (Affiliated Cancer Hospital of Chengdu Medical College), No. 1 Shi’er Zhong Street, Wuhou District, Chengdu, 610041 Sichuan Province China; 4 Department of Intensive Care Unit, Chengdu Seventh People’s Hospital (Affiliated Cancer Hospital of Chengdu Medical College), No. 1 Shi’er Zhong Street, Wuhou District, Chengdu, 610041 Sichuan Province China; 5 Department of Medical Oncology, Guangxi Academy of Medical Sciences and the People’s Hospital of Guangxi Zhuang Autonomous Region, Nanning, 530016 Guangxi China

**Keywords:** Advanced cancer patient, Eastern cooperative oncology group performance status, Experience, Care needs, Qualitative phenomenology

## Abstract

**Background:**

Advanced cancer patients with good Eastern Cooperative Oncology Group (ECOG) performance status (score 0–1) are underrepresented in current qualitative reports compared with their dying counterparts.

**Aim:**

To explore the experiences and care needs of advanced cancer patients with good ECOG.

**Design:**

A qualitative phenomenological approach using semi-structured interview was employed. Data was analyzed using the Colaizzi’s method.

**Setting/Participants:**

Purposive sample of terminal solid cancer patients on palliative care aged 18–70 years with a 0–1 ECOG score were recruited from a tertiary general hospital.

**Results:**

Sixteen participants were interviewed. Seven themes were generated from the transcripts, including experiencing no or mild symptoms; independence in self-care, decision-making, and financial capacity; prioritization of cancer growth suppression over symptom management; financial concerns; hope for prognosis and life; reluctance to discuss death and after-death arrangements; and use of complementary and alternative medicine (CAM) and religious coping.

**Conclusions:**

Advanced cancer patients with good ECOG have distinct experiences and care needs from their dying counterparts. They tend to experience no or mild symptoms, demonstrate a strong sense of independence, and prioritize cancer suppression over symptom management. Financial concerns were common and impact their care-related decision-making. Though being hopeful for their prognosis and life, many are reluctant to discuss death and after-death arrangements. Many Chinese patients use herbal medicine as a CAM modality but need improved awareness of and accessibility to treatment options. Healthcare professionals and policy-makers should recognize their unique experiences and needs when tailoring care strategies and policies.

**Supplementary Information:**

The online version contains supplementary material available at 10.1186/s12904-024-01425-3.

## Key statements

**What is already known about the topic?**

• Even in their advanced stage, cancer patients with good ECOG performance status are capable of self-care and less reliant on care provided by other.

• Existing qualitative research mainly focuses on advanced cancer patients with poor ECOG, emphasizing pain management, emotional distress, and palliative care.

**What this paper adds?**

• Our findings reveal distinct experiences and care needs of advanced cancer patients with good ECOG performance status from their dying counterparts.

**Implications for practice, theory or policy**.

• Healthcare professionals should recognize and address the patient group’s distinct needs.

• Future research should further investigate their symptom trajectory, influencing factors, and care needs to fill the gap in their cancer journey.

• Policy-makers should develop tailored policies that consider good ECOG performance status.

**Supplementary Information:**

The online version contains supplementary material available at 10.1186/s12904-024-01425-3.

## Introduction

Cancer is a major global public health issue, which affects millions of people around the world and in China. There were estimated 19.3 million new cancer cases and 10 million cancer deaths globally in 2020 [1]. China accounted for 24% of newly diagnosed cases and 30% of the cancer-related deaths [2]. As treatments and early detection methods continue to advance, the number of people living with cancer are on the rise, whose life expectancy is extended. For instance, the five-year survival rate for cancer patients in China rose from 30.9% in 2003–2005 to 40.5% in 2012–2015 [3], highlighting the growing population of individuals living with cancer for extended periods.

Cancer staging is crucial in clinical oncology. It provides pivotal information on the extent and spread of the disease to guide treatment decisions and prognosis [4]. Advanced-stage cancer patients, typically characterized by extensive tumor growth and metastasis, often face more aggressive treatments and poorer prognoses compared with those in an earlier stage. However, advanced cancer patients with good physical performance status, commonly measured as an Eastern Cooperative Oncology Group performance status (ECOG PS) score, are often underrepresented in the current qualitative research. Instead, their counterparts with poorer ECOG PS who are usually in the final stage of disease are the focus of current research interests, who typically experience more severe symptoms, functional decline, and higher risks of adverse events [5, 6]. Researchers may opt not to recruit patients with good ECOG PS, who are more likely to continue working and engaging in daily activities and may be less accessible [7]. Also, this patient population may be neglected in favor of cancer patients nearing death because of limited research funding and resources [8].

In our clinical practice of palliative and hospice care, we often encounter cancer patients in the advanced stage with good ECOG PS (score 0–1). They impress us by demonstrating distinct physical and psychological experiences as well as varied care needs from those with poorer PS. This led us to hypothesize that despite the advanced stage of disease, cancer patients with good ECOG PS may have different experiences and care needs from their dying counterparts. Therefore, we devised this qualitative phenomenological study, which aimed to explore the experiences and care needs of advanced cancer patients with good ECOG PS. Our findings may shed light on some distinct characteristics of this underrepresented patient group and reveal novel areas potentially meaningful for future research. The evidence may find merit in supporting care-providers and decision-makers to more specifically tailor their strategies when caring for cancer patients in the advanced stage.

## Methods

### Study design

This was a phenomenological qualitative study. Semi-structured, in-depth interviews were conducted face-to-face with eligible participants to gather information about their lived experiences and care needs. The interviews continued until data saturation was achieved [9], at which point no new insights emerged from further interviews. Colaizzi’s method was employed to analyze the collected data [10].

### Ethical consideration and informed consent

The study was ethically approved by the Ethics Committee of Chengdu Seventh People’s Hospital (reference number AF-SOP-09-2.1). Written informed consents were signed with all participants.

### Setting

The present study took place at the Oncology Department of Chengdu Seventh People’s Hospital, a tertiary general hospital situated in Chengdu, Sichuan Province, Southwest China. As one of the national pilot centers for palliative and hospice care, this department offers both curative cancer treatments and palliative or hospice care to cancer patients across all stages of tumor progression.

### Research team

Our research team consisted of 8 members, including 6 oncologists and 2 nurses. Each researcher had a minimum of 5 years of clinical experience in oncology, with at least 2 years in palliative care. Additionally, all team members were trained in phenomenological qualitative methodology. None of the researchers held religious affiliations or had any known inclined theoretical or ethical perspectives. The principal investigator underwent training in face-to-face semi-structured interview.

### Participants

Participants were recruited from patients who received treatment and/or follow-up care at the Oncology Department between July and September 2022. The principal investigator screened potential participants based on the following inclusion and exclusion criteria:

Inclusion criteria: A patient should (1) be 18–70 yrs old with a stage IV cancer diagnosis of solid tumor and a life expectancy > 6 mo; (2) have an ECOG PS score of 0–1; (3) be currently undergoing palliative therapies, including but not limited to palliative chemotherapy and radiotherapy; (4) be aware of their diagnosis; (5) be able to participate in a 30 min in-depth interview; (6) possess sufficient cognitive capacity and verbal communication ability; and (7) provide consent to participate in the study and for the publication of findings.

Exclusion criteria: A patient was ineligible if they (1) were < 18 or > 70 yrs old; (2) had a non-cancer or non-solid tumor diagnosis, a tumor stage below IV, an ECOG PS score > 1, or a life expectancy < 6 mo; (3) were unaware of their diagnosis; (4) were receiving hospice or end-of-life care; (5) were physically unfit for an interview, as assessed by the interviewer; (6) had a known mental illness or exhibited insufficient cognitive and communication capacity; or (7) failed to provide consent.

### Sampling and data collection

Purposive sampling was employed to select participants. The primary investigator accessed candidates’ medical records on the hospital’s electronic medical record system, screening them against the inclusion and exclusion criteria. She then approached a potential participant and made a brief casual conversation to visually assess their physical and mental status, cognitive abilities, and communication skills.

If the candidate seemed suitable for further interview, the primary investigator explained the study’s objectives and process, and inquired if they were interested in participating. After obtaining informed consent, the primary investigator either initiated the interview immediately or scheduled it for a later time, typically within the next 72 h. For those with scheduled interviews, the interviewer reassessed the participant’s status at the time of the interview. If the participant was deemed unfit by the primary investigator, the interview would be canceled and the participant excluded.

Participants were interviewed individually in a designated room, without the presence of family members. If a participant displayed signs of physical discomfort or reluctance to continue, the interview would be discontinued and any incomplete interviews discarded.

The sampling process persisted until data saturation was reached [9], at which point the interviews no longer produced new analytical information.

### Semi-structured interview

The primary investigator conducted all semi-structured interviews to ensure consistency across the study. To prevent interviewer burnout and allow for timely verbatim transcription, no more than three participants were interviewed on a single day. Each interview was audio-recorded in its entirety.

Before delving into specific questions, the interviewer posed a grand tour question to guide the participant, typically phrased as “How do you feel today?” or “Could you tell me about how you feel recently?” Probing questions such as “Could you tell me more about the care/doctors/nurses?“, “Has the therapy made you feel better?“, and “What makes you think that?” were utilized to encourage participants to provide more detailed responses. The interviewer employed various techniques, such as rhetorical questioning, repetition, and response, to uncover the participants’ genuine feelings. Field notes were taken throughout the interviews to document the participants’ tone of voice, notable facial expressions, and body gestures. (Supplement 1)

Immediately following each interview, the audio recordings and notes were cataloged. Another investigator transcribed the recordings verbatim and verified the accuracy of transcriptions within 24 h after the interview. To ensure anonymity, the records and transcriptions were de-identified, with participants assigned numerical identifiers (P1, P2, etc.) in place of personal information. All patient data, recordings, and transcriptions were maintained with strict confidentiality.

### Rigour

To ensure the rigor of this study, several strategies were employed. The interviewer maintained neutrality throughout the interviews by refraining from expressing personal opinions or judgments. When unclear statements or feelings arose, the primary investigator sought clarification from the participants during the interview. In cases of disagreement among researchers, the team referred back to the interview transcriptions and, if necessary, sought further clarification from the participants.

## Findings

### Participants and interviews

In this study, we interviewed 16 participants aged 33–64 yrs (mean age, 55.1 yrs), including 7 females. The interviews had an average duration of 17.7 min (range, 10.5–42.5 min). All participants were medically insured and diagnosed with stage IV solid tumors. Half of the participants (8/16) were asymptomatic, while the remaining experienced mild symptoms. Among the participants, 15 expressed expectations with their current treatment to suppress tumor growth, and 4 of symptomatic patients expected it to alleviate symptoms. Nine participants reported using Chinese herbal medicine (CHM) as a complement to their ongoing treatment. Two participants reported holding religious beliefs, both identifying as Christians. Detailed sociodemographic and clinical information of the participants is in Tables 1 and 2.

**Table 1:** Sociodemographic information of participants (*N* = 16)

	P1	P2	P3	P4	P5	P6	P7	P8	P9	P10	P11	P12	P13	P14	P15	P16
Age, years	45–60	45–60	45–60	> 60	30–45	45–60	45–60	> 60	> 60	45–60	45–60	45–60	> 60	> 60	15–30	> 60
ECOG	2	1	1	0	1	0	1	2	1	0	0	1	2	1	2	1
Marital status	Married	Married	Married	Married	Single	Married	Married	Married	Married	Married	Married	Married	Married	Married	Married	Married
Offspring	4	1	0	0	0	2	1	3	2	1	1	2	1	2	2	2
Children	3	1	1 (Deceased)	0	0	1	1	1	1	1	1	1	1	1	1	1
Grandchildren	1	0	0	0	0	1	0	2	1	0	0	1	0	1	1	1
Education level	Primary school	Junior high	Senior high	Junior high	College	Junior high	Junior high	Junior high	Junior high	Junior high	Primary school	Junior high	Junior high	Junior high	Junior high	Junior high
Occupation	Peasant	Retired	Small business owner	Factory worker	Office worker	Peasant	Peasant	Retired	Retired	Retired	Peasant	Small business owner	Peasant	Peasant	Small business owner	Peasant
Household income (Yuan RMB)	< 5,000	< 5,000	< 5,000	< 5,000	5,000–10,000	5,000–10,000	5,000–10,000	5,000–10,000	5,000–10,000	< 5,000	< 5,000	5,000–10,000	5,000–10,000	5,000–10,000	5,000–10,000	5,000–10,000
Living with	Alone, occasionally with spouse	Spouse	Spouse	Spouse	Parents	Children	Spouse	Spouse	Spouse	Spouse	Spouse	Spouse, children	Spouse, child	Spouse, children	Parents, spouse, children	Spouse, children
Informal caregiver	Self	Self, spouse	Self, spouse	Spouse	Parents	Self, children	Self, spouse	Spouse	Self, spouse	Spouse	Spouse	Self, spouse, children	Spouse, child	Spouse, children	Self, parents, spouse	Self, spouse
Pay for care	Medical insurance	Medical insurance	Medical insurance	Medical insurance	Medical insurance	Medical insurance	Medical insurance	Medical insurance	Medical insurance	Medical insurance	Medical insurance	Medical insurance	Medical insurance	Medical insurance	Medical insurance	Medical insurance
Religion	Christian	None	None	None	None	None	None	None	None	None	None	Christian	None	None	None	None

ECOG, Eastern Cooperative Oncology Group performance status score

**Table 2:** Clinical data of participants (*N* = 16)

	P1	P2	P3	P4	P5	P6	P7	P8	P9	P10	P11	P12	P13	P14	P15	P16
Cancer diagnosis	Lung	Colon	Lung	Gastric	Gastric	Gastric	Lung	Gastric	Pancreas	Lung	Nasopharyngeal	Nasopharyngeal	Lung	Colon	Colon	Rectal
Time of diagnosis	Jan-19	Jan-20	Feb-20	Jan-20	May-22	Dec-21	Jul-22	Jul-20	Apr-22	Jan-16	Apr-18	Aug-20	Nov-21	Mar-22	Mar-22	Jan-21
Current cancer treatment	Chemotherapy	Chemotherapy, targeted therapy	Chemotherapy, targeted therapy	Chemotherapy, immunotherapy	Chemotherapy, immunotherapy	Chemotherapy, immunotherapy	Chemotherapy	Chemotherapy	Chemotherapy	Chemotherapy	None	Chemotherapy	Radiotherapy	Chemotherapy	Chemotherapy	Radiotherapy
Symptom	Shoulder pain	Fatigue	Fatigue	None	None	None	Coughing	Abdominal distention	None	None	None	Nasal bleeding	Dizziness	None	Abdominal distention	None
Expectation with care	Tumor suppression, symptom management	Tumor suppression	Tumor suppression	Tumor suppression	Tumor suppression	Tumor suppression	Tumor suppression	Tumor suppression, symptom management	Tumor suppression	Tumor suppression	Follow-up	Tumor suppression	Tumor suppression, symptom management	Tumor suppression	Tumor suppression, symptom management	Tumor suppression
Complementary and alternative medicine	CHM	CHM, moxibustion	CHM	CHM	Not reported	CHM, acupuncture	CHM	Not reported	None	CHM	Not reported	CHM, acupuncture	None	CHM	Not reported	Not reported

CHM, Chinese herbal medicine

### Findings from interviews

We extracted seven overarching themes from the interviews, as follows:

#### Theme 1: experiencing no or mild symptoms

The majority of participants reported experiencing either no symptoms or only mild symptoms during the interview period, which did not substantially impact their daily lives. However, a few participants recounted instances of severe symptoms, such as debilitating pain, that hindered their ability to engage in routine daily activities.

*“(I) feel fine. Almost nothing. Just a little coughing now and then.” - P7*.

*“No, I feel not bad… (I) only feel more tired than before every day. Nothing else.” - P8*.

*“My shoulder used to hurt really badly. I couldn’t lift my right arm… It hurt so badly that I’d rather die… Couldn’t do anything… (The pain) was relieved again after ascites extraction. Now it hurts a little some times but OK.” - P11*.

The most severe symptom was described by Participant 16, who had experienced abdominal distention, which was managed by ascites extraction:

*“My belly felt very full all the time… Better after the ascites was extracted. (I) couldn’t do anything then but can take care of myself again now, at least do some of my own things now.” (Smiled) - P16*.

#### Theme 2: independence in self-care, decision-making, and financial capacity

The participants exhibited a strong sense of independence in multiple aspects, including self-care, care-related decision-making, and financial capability for care expenses.

Rather than being heavily dependent on family members or other informal caregivers, the majority of participants were either fully or partially self-sufficient in managing their daily living activities:

*“I take care of myself. My husband helps sometimes especially when I’m hospitalized but I don’t like his cooking.” - P1*.

*“I can take care of myself. They (family members) are busy working every day. I cook my meals. Easier for me to choose what I want to eat.” - P4*.

*“My wife looks after me and I try to take care of my own daily living as long as I feel good enough.” - P10*.

Most participants seemed resolute in making their own decisions while having little trouble seeking input from a variety of sources:

*“… I make my own decisions… My nephew works for a pharmaceutical company… He suggests me to ask if I can test only some of the gene sequencing tests…” - P1*.

In some cases, they even exhibited resistance to external interference with their decision-making, especial concerning their treatment-related decisions:

*“This is my life. (I) should think for myself, whether (I) continue my treatment or stop.” - P3*.

Some participants mentioned that they paid for their treatments with their own savings and were hesitant to spend money from other family members or borrowed money for fear of becoming a burden:

*“For now (I) still have enough money (for the treatments). (My medical) insurance reimburse most of the expense… more than 90% reimbursed. I only pay less than 10%.” - P5*.

*“I pay for my own treatments… (I) don’t want him (his son) to spend his money. He’s not even married yet.” - P16*.

Some did not even quit working:

*“I go back to work sometimes but they (superiors at work) don’t require me. Most of the time I work at home. Only some easy assignments… They pay me the minimal salary… I understand. It’s not easy for them either. Already very kind of them to keep me like this.” - P15*.

#### Theme 3: prioritization of cancer growth suppression over symptom management

Notably, when asked about their primary expectations with their current care, 15 out of 16 participants expressed a desire for cancer growth suppression. This included all 8 asymptomatic participants and 4 of the mildly symptomatic patients:

*“I’ll take the PD-1 treatment (a targeted therapy) if my gene sequencing can find some new biomarker.” - P6*.

*“… to control its (tumor) growth. I don’t know how much longer… There might be a new drug or treatment. Who knows.” (Laughed) - P7*.

Only 4 symptomatic participants mentioned alleviating their symptoms:

*“My shoulder hurt really badly… After the ascites extraction, it didn’t hurt any more. It came back later and was relieved again after ascites extraction. Now it hurts a little some times but OK.” - P1*.

#### Theme 4: financial concerns

During the interviews, all participants mentioned financial concerns or burdens at some point, in some cases without any prompting from the interviewer. This was frequently associated with their decision to discontinue their current treatment. Notably, “running out of money” was identified as the primary reason for discontinuing treatment, which was emphasized more strongly than “not respond to therapy”:

*“I’ll go on with the therapies as long as (I) have money… My son told me just to keep getting treated. He borrowed 8,000 Yuan when (he) came back from Shanghai. But I don’t want him to borrow money… I don’t want to become a burden (for my family). (I) just stop my treatment when I run out of money.” - P4*.

*“… I’ll go on (with the treatment) as long as I can. Have to discontinue if no more money. What else can I do?” - P6*.

The availability of medical insurance reimbursement played a critical role in enabling them to afford ongoing treatment:

*“The disease is a heavy burden. I can still afford it now. My medical insurance reimburses most of it but still a heavy burden. I’ll just discontinue therapy when run out of money.” - P2*.

#### Theme 5: hope for prognosis and life

Despite being aware of their diagnoses, the participants demonstrated a sense of hope for the prognosis of their treatments and their future prospects in life:

*“I have no regret in life. My only unfinished business is to see my son getting married. I want to live to see it.” - P1*.

*“The targeted therapy worked very well for me. I’m still taking it. Hopefully, it will last long.” - P9*.

Instead of giving up, they tended to seek new potential treatments if a particular therapy had failed:

*“The doctor told me to take gene sequencing and see if some new biomarkers can be found after I stopped responding to the last medicine. I did and am waiting for the results now. (I) hope they can find a new one… Is there any other treatment I can use?” - P2*.

*“… (the tumor) grew and spread again after my last surgery. The doctor said that more surgeries may not do any more good but there are still other possibilities, like targeted therapy.” - P7*.

Interestingly, none of the participants brought up topics such as the desire for dying with dignity or the consideration of a Do-Not-Resuscitate (DNR) agreement.

#### Theme 6: reluctance to discuss death and after-death arrangements

Two specific questions were incorporated into the interviews to inquire whether participants had contemplated their own death and discussed after-death arrangements with their families. The responses were varied. Nearly half of the participants (7/16) reported that they had either never or rarely thought about death, nor had they discussed after-death arrangements with their family members:

*“I have never thought about it (death). Don’t want to… still a little afraid to talk about it.” - P5*.

*“It crosses my mind sometimes but I don’t think about it… (I) have never discussed (the after-death arrangements) with them (family). It’s not time yet.” - P8*.

In contrast, some other participants seemed open to think about and discuss them:

*“I did. That’s fine. I know I have it (cancer). They know I have it too. There’s no need to hide or fear. It’s pointless. Doesn’t help with anything. I talked about it (my death) with them (my family) once. They were kinda shy and almost cried. Then I stopped.” (Laughed) - P12*.

*“(I) discussed my after-death arrangements with my family already. Better to get prepared earlier. No one knows when the time will come.” - P13*.

#### Theme 7: use of complementary and alternative medicine and religious coping

Seven participants reported utilizing complementary and alternative medicine (CAM) modalities, which were primarily limited to Chinese herbal medicine, acupuncture, and massage. The main reasons for use of CAM were to assist in suppressing tumor growth and alleviating symptoms:

*“I took Chinese herbal medicine after chemotherapy to help with my nausea and vomiting. I also had acupuncture for my headache.” - P2*.

*“I went to see a traditional Chinese medicine doctor. He was famous for treating cancer with herbal medicine.” - P9*.

An interesting observation was that two participants cited engaging in religious activities as part of their coping strategies. Notably, one of them adopted her religious practices shortly after receiving her cancer diagnosis:

*“I began to believing in Christianity two years again… one month after I was diagnosed. The sisters (fellow believers) said that they would pray for me to heal… I enjoy the peace and joy.” - P2*.

*“Yes, I’m a Christian… I pray for my health and healing sometimes.” - P16*.

## Discussion

### Main findings

On this qualitative study, we sought to explore the experiences and care needs of advanced cancer patients with good ECOG PS, a population that has not been extensively studied. Understanding their unique perspectives is vital for optimizing care and ensuring that the patients’ needs are met in the continuum of cancer care. We were able to extract seven overarching themes from the narratives of our in-depth interviews with 16 participants, including their symptoms, sense of independence, treatment priorities, financial concerns, hope, reluctance to discuss death, and use of complementary and alternative medicine.

In the current study, we found most of our participants either asymptomatic or mildly symptomatic. This is consistent with previous studies about the relationship between performance status, symptom burden, and disease stage. The ECOG PS is widely used and essential measure of the functional capacity of cancer patient, which is shown to correlate with clinical outcomes, treatment tolerance, and survival [11]. Patients with good ECOG PS generally have no or limited disease-related restrictions, despite the advanced cancer stage. According to Cormier et al., several factors may contribute to their contrast with the poor PS patients, such as better overall health, effective symptom management, and more favorable tumor characteristics or response to treatment [12]. This practically differentiates the two sub-groups of advanced cancer patients not only in terms of their experiences of symptoms but their distinct care needs. Compared with their counterparts with poor PS, the better performing patients need less symptom management and psychological support and may have different care priorities, which echoes with our finding that the participants prioritized tumor suppression therapies over symptom management. It is important for care-providers to recognize the variability in symptom burden due to varied ECOG PS, monitor the symptom trajectory of a patient, and devise care strategies accordingly.

The second theme highlights the participants’ strong sense of independence in various aspects of their cancer journey, including self-care, care-related decision-making, and financial capacity. Their good ECOG PS may enable them to maintain functional autonomy and actively engage in daily activities and decision-making processes [11], who are either fully or partially self-sufficient in managing their daily living activities. This finding is significant as advanced cancer patients with good PS may require less assistance from family members and other informal caregivers. This autonomy in self-care can contribute to their overall quality of life and psychological well-being because maintaining independence is often considered essential in coping with cancer [13]. The participants’ resoluteness in making their own decisions while seeking input from various sources demonstrates their active engagement in their care process, which may lead to better treatment outcomes and satisfaction with care, as patients who participate in their care decisions often report feeling more empowered and in control of their lives [14]. It is vital for healthcare professionals to respect and support the patients’ autonomy by providing the necessary information and guidance for them to make informed choices about care [15]. Another aspect of independence was their financial capacity. According to Lentz and colleagues, having the financial resources to pay for care can alleviate some of the stress and burden associated with managing cancer and its treatments [16], which can further contribute to a patient’s sense of control and well-being during their care journey.

It is worth noting, however, that the participants in this study may not be representative of the broader population of advanced cancer patients because they were all medically insured, which covered a significant portion of their medical expenses. This reduced their out-of-pocket costs significantly, contributing to their sense of financial independence. The potential selection bias in our sample should be considered when interpreting our findings. The experiences of participants with medical insurance might not accurately reflect those without such coverage. Uninsured or under-insured patients may face substantial financial burden and stress relating to the costs of care [16]. Furthermore, financial ability to pay for care can play a crucial role in a patient’s decision-making, particularly when it comes to deciding whether to continue or discontinue treatment. Patients who are financially constrained may be more likely to consider discontinuing treatment, even if they are clinically eligible for and may benefit from the treatment [16], which was evident in our study. The financial burden of cancer treatment can lead to significant distress, prompting patients to weigh the benefits of treatment against the costs [17]. This may result in decisions not entirely aligned with their medical needs and preferences and potentially compromise their quality of care and clinical outcomes [18]. It is imperative that healthcare professionals should be attentive to a patient’s financial concerns, even when they perform well physically, and engage in open discussions about the costs of care and potential resources to support their decision-making.

The finding that advanced cancer patients with good ECOG PS prioritize cancer suppression therapies over symptom management was expectable. This preference can be easily understood according to Maslow’s hierarchy of needs where one would strive to fulfill their basic needs before addressing higher-order needs [19]. In our context, symptom management can be considered a basic need, as it addresses a patient’s physiological and safety concerns. Once the basic need is met, patients tend to shift their focus to higher-order needs, such as achieving the best possible cancer control and prolonging survival. In our case, the participants with good ECOG PS are mostly asymptomatic or mildly symptomatic. Their basic needs in terms of symptom management are relatively well-addressed. Therefore, they are more likely to prioritize such higher-order needs as cancer suppression for better clinical outcomes and maintain their functional status. This finding has significant implications for improving the prognosis of advanced cancer patients with good ECOG PS, who are generally more functional and experience fewer severe symptoms and as a result may be physically more tolerant to therapies, including aggressive or experimental treatments. This increased tolerance can enable clinicians to consider a broader range of treatment options, which may lead to better cancer control and improved their clinical outcomes, including better symptom management and prolonged survival. Additionally, these patients seem more driven to explore new and experimental treatment options. Their willingness to participate in clinical trials or seek innovative therapies may provide them with access to cutting-edge treatments with potentially benefits for their prognosis and survival [20]. As healthcare professionals, we should recognize this motivation and support them by providing information on clinical trials or innovative therapies.

Hope is known to have significant implications for advanced cancer patients, which may influences their emotional well-being, treatment decisions, and overall quality of life [21–24]. We found that the patients with good ECOG PS demonstrate hope for their prognosis and future prospects of life. Though being hopeful is generally beneficial for advanced cancer patients and may reinforce patient’s motivation to seek care positively, it must be noted that it can sometimes lead to unrealistic expectations or misguided decision-making [25]. This is particularly relevant in advanced cancer patients with good ECOG PS because their hopefulness and desire for aggressive treatment may lead them to misjudge their physical status and pursue therapies with limited efficacy or significant side effects [26, 27]. It becomes crucial for healthcare professionals to be aware of this potential pitfall and ensure that patients are properly informed about their prognosis, treatment options, and potential risks and benefits.

It was noteworthy that none of our participants brought up such topics as the desire for dying with dignity or the consideration of a DNR agreement. Their relatively better quality of life and sense of hopefulness could make them more focused on treatment and improving prognosis, rather than considering end-of-life decisions [23, 24]. Besides fear for death, another possible attributing factor is culture. Death and after-death arrangements are commonly thought of as negative topics or even taboos in Chinese culture, which are usually avoided especially when an individual is still living [28]. This resonates with Theme 6 where many participants were found reluctant to contemplate their own death and discuss after-death arrangements with their families. Their needs to be prepared for death, seek dignity in passing, and make arrangements after their deaths are expected to increase as they near the end of life [29]. Healthcare professionals should monitor their disease progression closely and offer support when needed.

Almost half of our participants (7/16) reported using CAM modalities, which were primarily limited to Chinese herbal medicine, acupuncture, and massage. The main reasons for employing CAM were to aid in suppressing tumor growth and alleviating symptoms. This finding highlights the diverse approaches that patients may take in searching for effective treatments, particularly where conventional therapies have limited efficacy or are associated with substantial side effects [11]. The limited diversity of CAM modalities suggests that the patients may lack awareness or accessibility to a broader array of CAM options, which is consistent with previous reports [30, 31]. Furthermore, despite the clinical trials to investigate the safety and efficacy of Chinese herbal medicine as a complement to mainstream cancer treatments, there is still insufficient evidence to establish CAM modalities for suppressing tumor growth. As a result, use of CAM should be approached with caution and mainly considered for purposes other than tumor treatment.

It is intriguing to find that two of the participants engaged in religious activities as part of their coping strategies because Chinese people are often believed to have a lower prevalence of religious beliefs and studies investigating religious coping in the Chinese population are scarce [32]. A main impression of the two religious patients was that they seemed to use religion for practical purposes, rather than fully embracing the beliefs. This is consistent with previous studies where cancer patients may seek out religious practices as a way to manage stress, find peace, and maintain a sense of hope during difficult times. Praying for healing, for example, can provide a sense of control and agency in a situation where they may feel powerless [33]. Future research may continue to pursue the subject among Chinese cancer patients.

Compared with dying advanced cancer patients with poor ECOG PS, those with good ECOG PS display distinct experiences and care needs. They generally have milder symptoms, higher independence in self-care, decision-making, and financial capacity, and prioritize tumor suppression therapies over symptom management. On the other hand, patients with poor ECOG PS grapple with a higher symptom burden, increased reliance on support, and a focus on symptom relief and palliative care. Though sharing concerns about finances, demonstrating hopefulness, utilizing CAM, the two groups have varying degrees and objectives. Recognizing these distinctions is essential for healthcare professionals to provide customized, patient-centered care to address the unique needs of the well-performing cancer patients.

### Strengths and limitations

As one of the few qualitative investigations to explore the experiences and care needs of advanced cancer patients with good ECOG PS, our study revealed that this patient group is distinct from their dying counterparts, who are the focus of the current research literature. Our findings highlight some observations characteristic of the patient group such as their asymptomatic or mildly symptomatic experiences, stronger sense of independence in various perspectives, and distinct care prioritization from those nearing death. However, further quantitative studies are needed to determine the association and synergistic dynamics of such characteristics.

### Implications for practice

It is vital for healthcare professionals and policy-makers to recognize these unique experiences and care needs of advanced cancer patients with good ECOG PS and respond by providing necessary information, education, treatment options, and care strategies.

## Conclusions

Advanced cancer patients with good ECOG have distinct experiences and care needs from their dying counterparts. They tend to experience no or mild symptoms, demonstrate a strong sense of independence, and prioritize cancer suppression over symptom management. Financial concerns were common and impact their care-related decision-making. Though being hopeful for their prognosis and life, many are reluctant to discuss death and after-death arrangements. Many Chinese patients use herbal medicine as a CAM modality but need improved awareness of and accessibility to treatment options. Healthcare professionals and policy-makers should recognize their unique experiences and needs when tailoring care strategies and policies.

### Electronic supplementary material

Below is the link to the electronic supplementary material.

Supplementary Material 1

## Data Availability

Due to the sensitive nature of the interview recordings, the original audio recordings are prohibited from sharing. The desensitized transcripts and demographic data are available from the corresponding author on reasonable request.
