# Supplementary material for: Distinct experiences and care needs of advanced cancer patients with good ECOG performance status: a qualitative phenomenological study
Source: BMC Palliat Care. 2024 Apr 17;23:102. doi: 10.1186/s12904-024-01425-3 (PMC11022448; doi:10.1186/s12904-024-01425-3)
Supplement: Supplementary file 1 — Supplementary Material 1 [file 12904_2024_1425_MOESM1_ESM.docx]

Interview Design: Semi-structured Interview

Interview Location: Beside the patient's bed in the ward, preferably in a single-patient room with the patient's family waiting outside.

Interview Duration: Approximately 30 minutes.

Interview Recording:

(1) Notes: Key points, significant expressions and tone, emotional changes.

(2) Audio Recording: 1-2 sets of recording devices.

Interview Content and Techniques:

(1) Introduction:

a. Greetings and brief conversation to create a relaxed research atmosphere.

b. Begin by explaining the purpose of the study and the interview content. Specifically inform the patient about the following points:

1) Our research is not directly related to your medical condition or recent treatment.

2) During the interview, we may touch upon sensitive topics such as religious beliefs, family relationships, treatment costs, and even attitudes towards death. These are all part of our research and have no direct connection to your ward or treatment.

3) One of the main objectives of this study is to analyze the interview results of you and other patients to help us gain a better understanding of the impact of the disease and our treatment from the perspective of patients. This will enable us to improve the quality of our medical services and better assist patients in achieving better treatment outcomes. Additionally, by analyzing the interview results, we will write and publish scientific research papers to share our findings with the world. Therefore, your participation is very important and valuable.

4) The upcoming interview will cover the following aspects:

a. Your evaluation and feelings about the current treatment as a patient.

b. Changes in your life before and after being ill, including the emotional journey, family relationships, daily life, emotions, work, income, and religious beliefs before and after being diagnosed.

c. Your views on death.

(2) Interview Questions:

a. Opening Questions: (These questions can be simplified if there is limited time)

1) According to your medical records, were you diagnosed with XXX (type of cancer) at the time of (diagnosis time)?

2) How did you learn about our hospital?

3) When did you start receiving treatment at our hospital?

4) Why did you choose our hospital?

b. Recent Treatment Experience:

1) How have you been feeling during the recent period of treatment? What specific changes have you noticed in your physical condition?

2) Is there anything specific that you would like our doctors or nurses to know but haven't been able to tell us yet?

3) Can you recall any particularly memorable experiences during your recent treatment? What do you think has been good or bad?

4) How have you been feeling emotionally recently?

c. Changes Before and After Falling Ill:

1) (Continuing from the previous question about emotional changes) Do you remember your usual emotional state before you became ill? Have there been any changes since the diagnosis? (Based on the patient's answer, follow up with questions to understand their entire emotional journey after the diagnosis, especially for patients who have been ill for a long time.)

2) Have you shared these inner feelings with your family? (This is a transitional question; regardless of the answer, continue with the next question) Who do you currently live with? Who primarily takes care of you and accompanies you for medical visits? Have you noticed any changes in the people around you since you became ill?

3) Do you feel a significant financial burden from the treatment now? Can your treatment expenses be reimbursed? Approximately what percentage can be reimbursed? Who is primarily responsible for paying your treatment expenses?
